# ID 273 - Cálculo Automatizado de Efeitos Absolutos por meio do Programa Abseff: estudo descritivo

**DOI:** 10.5327/2237-9622.2025.v34s1.273

**Published:** 2025-11-25

**Authors:** Isabela Porto de Toledo, Rafael Leite Pacheco, Ana Luiza Cabrera Martimbianco, Carolina de Oliveira Cruz Latorraca, Veronica Colpani, Isabela Porto de Toledo, Roberta Borges Silva, Camila Cruz Monteiro, Cecília Menezes Farinasso, Patrícia do Carmo Silva Parreira, Rachel Riera

**Keywords:** síntese de evidências, efeitos absolutos, medidas de tamanho de efeito, tradução do conhecimento, Stata, R

## Abstract

**Introdução:**

Uma das formas de se comunicar o efeito de intervenções é por meio da conversão de efeitos relativos, como, por exemplo, o risco relativo, em medidas de efeito absoluto. Recomendações do GRADE (do inglês, ) e do sugerem a conversão das medidas de efeito relativo para unidades naturais nos resumos e nas conclusões das sínteses de evidências. Essa conversão pode levar a erros, e investigações anteriores já indicam que inadequações são frequentes em revisões Cochrane que convertem para medidas de efeito absoluto.

**Método:**

Estudo descritivo da elaboração do programa abseff desenvolvido na linguagem de programação do software Stata e em R. Esse programa foi desenvolvido no contexto de dois projetos conduzidos pelo Núcleo de Avaliação de Tecnologias em Saúde/Núcleo de Evidências do Hospital Sírio-Libanês (Nats/NEv-HSL) em colaboração com o Ministério da Saúde, a Agência Nacional de Saúde Suplementar e o Conselho de Nacional de Justiça por meio do Programa de Apoio ao Desenvolvimento Institucional do Sistema Único de Saúde (Proadi-SUS). Ambos os projetos envolvem a elaboração de sínteses de evidências, e o desenvolvimento do abseff visa à conversão de medidas de efeito relativo em medidas de efeito absoluto.

**Resultados:**

O programa abseff funciona por meio da coleta de dados de efeito relativo da intervenção para a conversão de medidas de efeito absoluto. Foram incorporadas fórmulas para conversão de risco relativos, e . O abseff também calcula os intervalos de confiança de 95% de todas as estimativas. De modo geral, o pesquisador precisa escolher a medida de efeito relativo e o intervalo de confiança, assim como um risco basal que será utilizado para o cálculo. Um exemplo de seria abseff rr 0.83 0.63 1.03 50 130. Nesse caso, está se solicitando a conversão de um risco relativo de 0.83 (IC 95% de 0.63 a 1.03), considerando um risco basal de 50/130 (38%). O resultado é apresentado tanto em efeitos absolutos antecipados quanto na diferença de risco absoluto. Também são consideradas as bases 100 e 1.000 em todos os cálculos. O programa é de download gratuito via Stata e R, e mais detalhes podem ser encontrados em: https://rlpacheco.github.io/abseff/ e https://github.com/rlpacheco/abseff_R .

**Conclusão:**

A comunicação efetiva de evidências pode ser desafiadora, e a tradução do conhecimento científico pode ser facilitada ao se apresentar medidas de efeito absoluto ao tomador de decisão. O abseff possibilita uma padronização dos cálculos, evita erros e garante a reprodutibilidade das análises. O abseff foi uma ferramenta criada no âmbito de dois projetos financiados pelo Proadi-SUS, mas que poderá ser utilizado de maneira aberta por todos os Nats da Rede Brasileira de Avaliação de Tecnologias em Saúde (Rebrats).

